# Antidepressant responsiveness in adulthood is permanently impaired after neonatal destruction of the neurogenic pool

**DOI:** 10.1038/tp.2016.255

**Published:** 2017-01-03

**Authors:** S Yu, I Zutshi, R Stoffel, J Zhang, A P Ventura-Silva, N Sousa, P S Costa, F Holsboer, A Patchev, O F X Almeida

**Affiliations:** 1 Max Planck Institute of Psychiatry, Munich, Germany; 2 Max-Delbrück-Zentrum for Molecular Medicine in the Helmholtz Association, Berlin, Germany; 3 Life and Health Sciences Research Institute (ICVS), University of Minho, Braga, Portugal; 4 ICVS/3B’s—PT Government Associate Laboratory, Braga/Guimarães, Portugal; 5 HMNC Brain Health, Munich, Germany; 6 Brain Research Instrument Innovation Center, Suzhou Institute of Biomedical Engineering and Technology, Chinese Academy of Sciences, Suzhou, China

## Abstract

The dynamic turnover of hippocampal neurons is implicated in the regulation of cognitive and affective behavior. Extending our previous demonstration that administration of dexamethasone (ND) to neonatal rats depletes the resident population of neural precursor cells (NPC) and restrains the size of the neurogenic regions, we now show that the adverse effects of ND persist into adulthood. Specifically, ND impairs repletion of the neurogenic pool and neurogenesis; ND also compromises cognitive performance, the ability to actively adapt to an acute stressor and, the efficacy of glucocorticoid (GC) negative feedback. Interestingly, although ND depletes the neurogenic pool, it does not permanently abolish the proliferative machinery of the residual NPC population; however, ND increases the susceptibility of hippocampal granule neurons to apoptosis. Although the antidepressant fluoxetine (FLX) reverses the latter phenomenon, it does not replenish the NPC pool. Treatment of ND-treated adult rats with FLX also improves GC negative feedback, albeit without rescuing the deleterious effects of ND on behavior. In summary, ND leads to protracted disruption of mental functions, some of which are resistant to antidepressant interventions. We conclude that manipulation of the NPC pool during early life may jeopardize the therapeutic potential of antidepressants in adulthood.

## Introduction

The dynamic acquisition and loss of hippocampal neurons is implicated in the regulation of mood, cognition and the neuroendocrine response to stress.^1, 2, 3^ The subgranular zone (SGZ) of the hippocampal dentate gyrus is endowed with a pool of neural precursor cells (NPC) that proliferate and differentiate into neurons or glial cells.^4^ Integration of these newly formed cells into the existing hippocampal circuitry influences cognitive performance^1, 2, 4, 5^ as well as affective behavior.^6, 7, 8, 9, 10^ Neurogenesis persists throughout life but is subject to negative modulation by intrinsic and extrinsic factors such as age,^11, 12^ stress^13, 14^ and high glucocorticoid (GC) levels.^15, 16, 17^

The GC receptor agonist dexamethasone (DEX) is often used to treat life-threatening conditions in perinatal medicine despite concerns regarding optimal dosage and potential adverse effects.^18, 19^ The latter concerns arise from preclinical and clinical reports that perinatal DEX treatment can severely retard psychomotor, emotional and cognitive development.^20, 21, 22, 23^ As high levels of GC are encountered during stressful events, it is pertinent to note that clinical studies have shown that early life experiences have a critical role in shaping an individual’s mental health span trajectory.^24, 25, 26, 27^

The present study involved broad behavioral phenotyping of adult rats that had received DEX during early postnatal life (neonatal DEX, ND), with a focus on emotional and stress-coping behavior, and hippocampus-dependent spatial memory. As hyperactivity of this axis is causally linked with impaired learning and memory^3^ as well as the ability to elicit adaptive behaviors that promote health and survival,^28, 29^ the impact of ND treatment on the activity of the hypothalamo–pituitary–adrenal (HPA) axis was also monitored. The decision to pay particular attention to emotional and cognitive performance during adulthood was based on our earlier demonstration that ND lastingly depletes the neurogenic pool and retards volumetric growth of the rat dentate gyrus.^30^ Lastly, given that antidepressants increase hippocampal neurogenesis^31, 32^ and concomitantly reverse some of the anomalous behaviors induced by stress during adulthood,^7, 8, 33^ we also examined the potential of fluoxetine (FLX), a commonly-used antidepressant, to ameliorate the undesired effects of ND exposure.

## Materials and methods

### Experimental subjects

Experiments were carried out on litters derived from 13 Wistar dams (Charles River, Sulzfeld, Germany), culled to 10 pups per litter at birth. On weaning (21 days) animals from different litters were randomly assigned to new housing groups (*n*=5 per cage). Procedures on animals were approved by the Regierung von Oberbayern and complied with European Union Directive 2010/63/EU. Throughout, animals were exposed to light from 0600 to 1800 (ZT0-ZT18), and all training and behavioral test sessions occurred between 0900 and 1200 (ZT3-ZT6). Group sizes for behavioral and morphological experiments were decided on the basis of pilot experiments on pup and adult rats (see figure captions for exact group sizes).

### Neonatal dexamethasone

Male pups received s.c. injections of vehicle (saline) or DEX (Fortecortin, Merck, Darmstadt, Germany) on postnatal day (PND) 1–7 (DEX 200 μg kg^−1^ day^−1^ on PND 1–3, tapered down to 100 μg kg^−1^ day^−1^ on PND 4–7); the neonatal vehicle- and DEX-treated groups are hereafter referred to as CON and ND, respectively. Animals that showed signs of weakness or discomfort were immediately culled in accordance with rules on animal welfare.

### Fluoxetine treatment

At 3 months of age, animals were housed individually for at least 1 week before receiving daily i.p. injections of either saline or FLX (10 mg kg^−1^; Kemprotech) for 4 weeks (Supplementary Figure S1A).

### HPA axis function tests

At end of the study (week 16), blood samples (~100 μl) were withdrawn at the daily nadir (ZT3) and peak (ZT18) of corticosterone (CORT) secretion; harvested serum was assayed for CORT by radioimmunoassay (MP Biochemicals, Costa Mesa, CA, USA). Blood samples were obtained after venepuncture of the dorsal tail vein, which was then sealed by application of light pressure before returning the animals to their home cages; during this whole procedure (<2 min), rats were lightly restrained with a soft towel. Animals were handled, restrained and underwent sham-venepunctures on 2–3 occasions before collection of experimental test probes.

The response of the HPA axis to an acute psychological stressor (air-puff)^34^ was tested between ZT3 and ZT5. Tail blood samples, collected at 0 min and at 30 and 120 min thereafter, were subsequently assayed for CORT.

The dexamethasone suppression test (DST)^35^ was used to examine the efficacy of negative feedback regulation of the HPA. A bolus of DEX was administered at ZT12 and tail-blood samples were collected for CORT measurements at ZT18.

### Behavioral phenotyping

**Figure 1:**
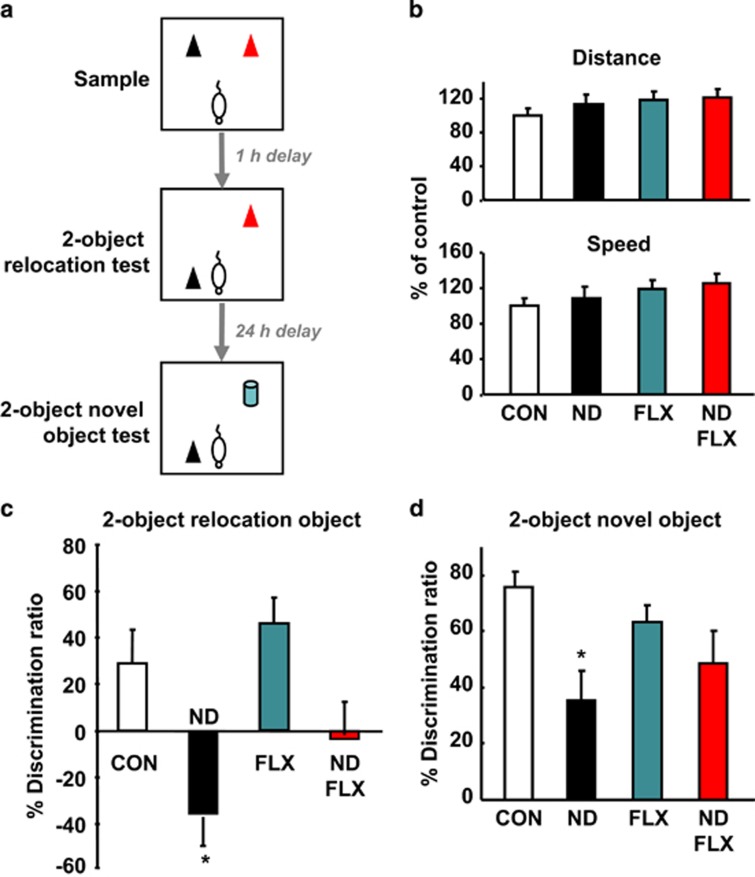
Spatial and novel object-recognition memory are impaired after neonatal dexamethasone (ND) exposure and fluoxetine (FLX) cannot rescue these cognitive deficits. The schematic (**a**) summarizes the paradigms used to assess two object-recognition memory. Discrimination between the objects was determined using a discrimination ratio, calculated as the difference in time spent exploring the novel object vs the familiar object divided by the total time spent exploring both objects.^36^ Initially, the distance traveled and speed of movement of all experimental groups was assessed in the open field arena without objects; the results are shown in **b**. Results of the 2-object relocated object preference test are shown in **c**. This test revealed that ND-treated rats are impaired in their ability to discriminate between a relocated object and an object in its original location, and that this impairment of spatial recognition is not restored after treatment with FLX. Examination of novel object preference (**d**) revealed that ND are retarded in their ability to discriminate between a novel and familiar object, and that this impairment is cannot be rescued with FLX. Data are mean±s.e.m. (*n*=13, 13, 13 and 12 for CON, ND, FLX and ND–FLX groups, respectively). *P<0.05 vs control.

Recognition memory (novel object-recognition test; Figure 1a),^36^ anxiety (open field (OF) and elevated plus maze (EPM) tests),^37, 38^ and capacity to adopt an active and adaptive behavior during acute stressor (forced-swim test)^33, 39^ were tested in rats (CON: *n*=26; FLX: *n*=26; ND: *n*=13; ND–FLX: *n*=12). Tests were performed between ZT3 and ZT6, with intervals of several days between each test to avoid carry-over effects; daily vehicle and FLX injections were administered after each behavioral test (Supplementary Figure S1A). Video-recorded behaviors were scored using ANY-Maze software (Stoelting, Kiel, WI, USA) in a blinded manner.

### Neonatal hippocampal NPC cultures and treatments

Primary neuronal cultures from the hippocampi of 4-day-old (P4) Wistar rats were prepared as previously described.^30^ DEX was added to cultures (10−6 M, 24 h) after 6 days *in vitro* (DIV). Cells were then washed with phosphate-buffered saline and re-incubated in culture medium; in some cases, cultures were treated with DEX (10−6 M) for a further 48 h.

### Immunostaining

At the end of the experiment, rats (aged 4 months) were anaesthetized, perfused with paraformaldehyde (4%) and killed; brains carefully excised, cryo-preserved and stored at −80 °C until sectioning (20 μm serial coronal cryosections over the whole length of the hippocampal formation, keeping every 10th section) (Supplementary Figure S1A). Sections were then sequentially incubated with antisera against Ki67 (1:500, DAKO, M7248), Sox2 (1:300, Santa Cruz; sc-17320) and cleaved caspase 3 (1:200, Cell Signaling, Danvers, MA, USA/NEB; #9661). Immunoreactivity was visualized using appropriate Alexa Fluor-conjugated secondary antibodies (Invitrogen). Volumes of the different subdivisions of the dentate gyrus and cell densities (*N*_V_) in the SGZ (defined as a two-cell layer-thick zone on the inner side of the granule cell layer of the dentate gyrus) were estimated (by an investigator blind to the treatment).^8^ The total number of SGZ cells of a given chemophenotype was derived from the product of Nv and SGZ volume. Immunostaining of BrdU-incorporating NPC *in vitro* was performed and analyzed as described previously.^30^

### Immunoblotting

Hippocampi, obtained from subgroups of animals at the end of the experiment (*n*=3 per group) were lysed and immunoblotted^40^ to detect Bax (1:1000, EMD Millipore, Darmstadt, Germany; 04-434), Bcl-xl (1:1000, Abcam, Cambridge, UK; ab32370) and Bcl-2 (1:1000, BD Biosciences, San Jose, CA, USA; 554087), using an enhanced chemiluminescence detection kit (GE Life Sciences, Freiburg, Germany). Blots were scanned, checked for linearity of signal and quantified (TINA 3.0 Bioimaging software, Raytest, Straubenhardt, Germany) after subtraction of local background. Normalized data are expressed as a percentage of controls.

### Statistics

Appropriate sample sizes were determined on the basis of previous experiments in our laboratory (effect size *f*=1, type I error [*α*]=0.05 and minimum level of statistical power [1−*β*]=0.8). Numerical data (mean±s.e.) were subjected to 2-tailed Student *t*-tests or analysis of variance and appropriate *post-hoc* analysis (IBM SPSS Statistics for Windows, Version 22.0; IBM, Armonk, NY, USA). Two-way ANOVA, followed by appropriate *post-hoc* tests were used to determine the effects and interactions of the ND and FLX treatments.

## Results

### Persistent, fluoxetine-irreversible depletion of the NPC pool by ND

The efficacy of the DEX and FLX treatments are shown in Supplementary Figure S1. Confirming that ND retards the volumetric growth of the SGZ and granule cell layer (GCL),^30^ stereological analysis showed that ND exerts inhibitory effects on hippocampal growth and the SGZ (*P*=0.03, Supplementary Figure S2A) and GCL volume (*P*=0.03; Supplementary Figure S2B), effects that persist through to adulthood.

**Figure 2:**
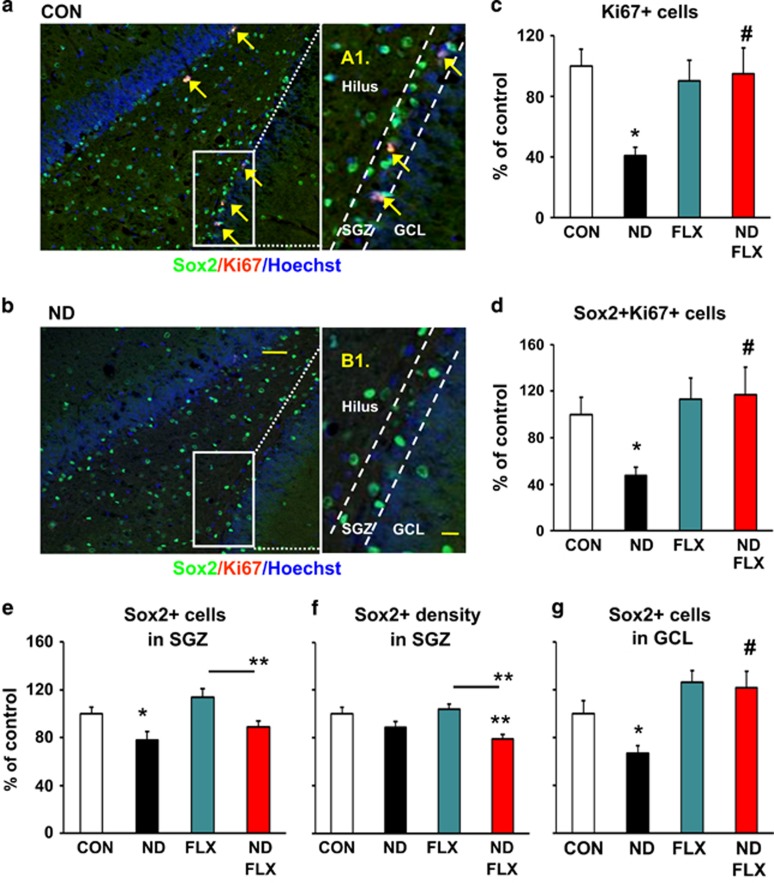
Persistent and fluoxetine (FLX)-irreversible depletion of the neurogenic pool in the subgranular zone (SGZ) of the hippocampus. (**a** and **b**) Representative confocal images of the SGZ in the adult control (CON, **a**) and ND-treated (ND, **b**) rat; sections were double-stained for Sox2 (neural precursor cells (NPC)) and Ki67 (mitotic cells) as well as Hoechst 33342 (cell nuclei); Sox2- and Ki67-immunopositive are labeled with arrows. The dotted lines indicate arbitrary demarcation of the SGZ (defined as three-cell layer zone at the granule cell layer (GCL)-hilus border; the insets in **a** and **b** are enlarged in A1 and B1, respectively. Scale bars in **a, b** and A1, B1 are 50 μm and 10 μm, respectively. (**c**–**e**) The effects of ND, FLX and ND–FLX treatment on the number of Ki67-, Sox2/Ki67- and Sox2-positive cells in the SGZ at the time of killing. There was an ~50% reduction in the number of proliferating cells (**c**)/proliferating NPC (**d**) in the SGZ of ND animals, an effect that was reversed in a subgroup of ND-treated animals that were treated with FLX during adulthood. Note the ~22% reduction in the number of NPC (**e**) in the SGZ of adult animals that had been exposed to ND, and the significantly lower number of NPC in the ND–FLX subgroup, as compared with the corresponding FLX group. (**f**) The effects of ND, FLX and ND–FLX treatments on the density of NPCs (Sox2+) in the SGZ. It is important to note that, FLX failed to restore SGZ Sox2+ cell density to control levels when the influence of volumetric effects of FLX (cf. Supplementary Figure S2A) is considered. (**g**) ND reduces the number of migrating neural progenitors (judged by number of Sox2+ cells in GCL), an effect that can be rescued by FLX during adulthood. Numerical data are mean±s.e.m. (*n*=8 for all groups). *indicates *P*<0.05 as compared with CON (**c**–**g**) or pairs of treatment groups (**e** and **f**). ^#^indicates *P*<0.05 vs corresponding ND-treated groups.

The SGZ harbors a pool of NPC which was markedly depleted by ND. Specifically, ND reduced the number of Sox2+ (NPC) cells (*P*=0.02; Figures 2a–c and e), Ki67 (mitotic) cells (*P*=0.002; Figures 2a–c) and Sox2+/Ki67+ (proliferating NPC) cells (*P*=0.04; Figures 2a–d) in the SGZ. Further, ND was found to disrupt the migration and maturation of NPC from the SGZ to the inner GCL (cf. Zhao *et al.*^4^), evidenced by the significant reduction in the number of Sox2+ cells within the GCL of ND-treated animals (*P*=0.04; Figure 2g).

Antidepressants, including FLX, stimulate neurogenesis, thus, possibly contributing to the therapeutic effects of antidepressants.^31^ There was a significant interaction between ND and FLX with respect to Ki67-positive proliferating cells (F_1,32_=6.45, *P*=0.02), but not Sox2-positive NPCs (F_1,32_=0.06, *P*=0.81), in the SGZ; specifically, FLX significantly increased the number of proliferating cells (Ki67+: ND vs ND–FLX, *P*=0.005; Figure 2c) and proliferating NPCs (Sox2+/Ki67+: ND vs ND–FLX, *P*=0.008; Figure 2d) in the SGZ of ND-treated rats. Notably, FLX did not influence the number of proliferative cells among any of the sub-populations of cells examined in control animals (Ki67+: control vs FLX, *P*=0.59; Sox2+Ki67+: control vs FLX, *P*=0.60; Figures 2c and d).

The above effects of FLX were accompanied by a corresponding increase in the number of migrating (Sox2+) NPCs in the GCL of FLX-treated vs untreated ND-exposed rats (ND vs ND–FLX, *P*=0.001; Figure 2g). However, although FLX did not significantly increase the number of NPCs in the SGZ of ND-exposed rats (Sox2: ND vs ND–FLX, *P*=0.23; Figure 2e), the number of NPCs in the ND–FLX group was significantly lower than that found in the SGZ of control (non-ND) rats that received FLX (Sox2: FLX vs ND–FLX, *P*=0.008; Figure 2e).

Administration of FLX to ND-treated animals leads to a recovery of the SGZ volume to a level found in control animals; as compared with the vehicle-treated ND group, the volume of the SGZ was greater in ND animals that received FLX (ND vs ND–FLX, *P*=0.0004) (Supplementary Figure S2A). This, together with the finding that FLX does not restore the density of NPCs in the SGZ to control levels (F_3,31_=5.50, *P*=0.004; control vs ND–FLX: *P*=0.005; Figure 2f), adds support to our previous report on the irreversible depletion of NPC pool by ND treatment.^30^

### DEX blocks cell proliferation in a reversible manner

The question of whether ND alters the proliferation rate of NPC was approached in primary hippocampal NPC cultures that were treated with DEX (10−6 M, 24 h). Predictably,^41, 42, 43^ exposure to DEX (24 h) reduced the number of BrdU-positive cells by ~40% (cf. Supplementary Figures S3A and C with Supplementary Figure S3E; *P*=0.01, compared with control cells). Following the withdrawal of DEX, the number of BrdU-incorporating cells increased to levels found in control cultures within 24 h (*P*>0.05 vs control; Supplementary Figure S3E).

Previous studies demonstrated that DEX blocks neural cell proliferation by inducing arrest in the G1 phase of the cell cycle.^41, 43^ As depicted in Supplementary Figures S3F and G, withdrawal of DEX from hippocampal NPC cultures alters the expression of two key regulators of the cell cycle, cyclinD1 (up-regulated) and p27 (down-regulated). Notably, following the withdrawal of DEX, good temporal coincidence was found between the expression patterns of cyclinD1 and p27 and the increased BrdU incorporation (Supplementary Figures S3E–G; CycD1-DEX vs CycD1-CON at 4 h, *P*>0.05, and p27-DEX vs p27-CON at 24 h, *P*>0.05). These *in vitro* findings provide a first mechanistic insight into how ND depletes the resident NPC pool and prevents replenishment of the granule cell population by agents such as FLX (cf. Figure 2f).

### Extended susceptibility of NPC population to apoptosis after ND treatment

**Figure 3:**
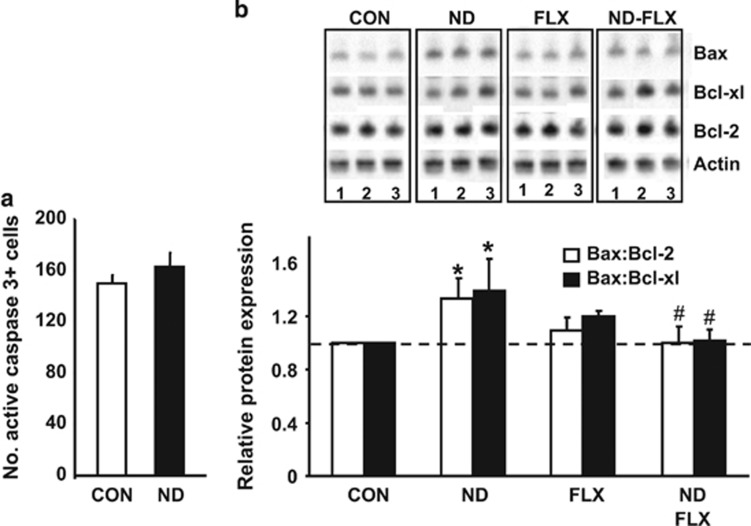
Fluoxetine (FLX) attenuates neonatal dexamethasone (ND)-induced susceptibility of hippocampal cells to apoptosis. (**a**) The number of activated caspase 3-positive cells in the subgranular zone (SGZ) did not differ between control and ND-treated rats; stereological estimates were made at the end of the experiment (cf. Figure 2). (**b**, *upper*) Western blots showing the influence of ND and/or FLX on the regulation of Bcl-2 family members in the hippocampus of adult rats; the *lower* panel presents the semi-quantitative results of the immunoblotting analysis (*n*=4 replicates). Note the increased expression ratios of Bax (pro-apoptotic molecule) to Bcl-2 or Bcl-xL (anti-apoptotic molecules) in the hippocampi of ND-treated rats and the restoration of these ratios to those found in controls by FLX. Numerical data represent mean±s.d. *P<0.05 vs control; ^#^*P*<0.05 vs ND.

Apoptotic events were previously implicated in ND-induced depletion of the neurogenic pool during early postnatal development.^30, 44, 45^ The results shown in Figure 3 show persistent upregulation of the apoptotic machinery in adult ND-treated animals: the hippocampi of ND-exposed rats displayed significantly higher expression ratios of the pro-apoptotic protein Bax vs the anti-apoptotic proteins Bcl-xL and Bcl-2 (cf. ref. 44 (control vs ND: *P*=0.003 and *P*=0.005, for the ratios of Bax:Bcl-xL and Bax:Bcl-2, respectively)), albeit without a contemporaneous increase in the number of activated caspase 3-positive cells (Figure 3a). Two-way ANOVA revealed a significant interaction between ND and fluoxetine with respect to the expression ratios of the pro- and anti-apoptotic molecules, Bax and BCl2 and Bcl-xL (Bax:Bcl-xL, F_1,103_=33.29, *P*=0.000; Bax:Bcl-2: F_1,117_=5.6, *P*=0.02). Although FLX did not significantly influence these ratios in control rats (control vs FLX: *P*=0.7 for Bax:Bcl-xL; *P*=0.61 for Bax:Bcl-2), the antidepressant reversed them in ND-treated animals (Bax:Bcl-xL: ND vs ND–FLX *P*<0.001; Bax:Bcl-2 ND vs ND–FLX *P*=0.001) (Figure 3b).

### ND-induced cognitive impairment is FLX-irreversible

Inhibition of hippocampal neurogenesis is known to impair spatial discrimination^5^ and spatial recognition memory.^46^ In experiments to examine the impact of ND-induced depletion of NPC in the novel object-recognition test, ND and FLX did not significantly influence exploration time in either the sample (F_3,48_=0.98, *P*=0.41), relocated object test (F_3,40_=0.65, *P*=0.59) or novel object test (F_3,37_=0.19, *P*=0.9; Figure 1a) phases. None of the groups differed in terms of locomotor activity (total distance traveled and speed) (F_3,48_=1.9, *P*=0.14 and F_3,48_=1.9, *P*=0.14, respectively), as measured in an OF arena (Figure 1b).

Analysis of results obtained in the spatial recognition test revealed main effects of ND (F_1,37_=12.43, *P*=0.001) but not of FLX (F_1,37_=2.55, *P*=0.12); no significant interaction effects of ND and FLX were detected (F_1,33_=0.28, *P*=0.6). As shown in Figure 1c, whereas control animals orientated to the new location (expected), ND-treated rats rigidly preferred the object in its familiar location (discrimination ratio in controls vs ND-treated rats: *P*=0.007). No differences were found between FLX-treated and control rats in the spatial recognition test (control vs FLX: *P*=0.48). Further, FLX did not improve the deficit in spatial memory displayed by ND animals (ND vs ND–FLX: *P*=0.12; Figure 1c) despite the efficacy of FLX in stimulating hippocampal neurogenesis (cf. Figures 2d and e).

Examination of recognition memory that depends on structural integrity of the hippocampus^47^ (Figure 1a) showed that ND-treated rats were significantly impaired in their ability to differentiate between novel and familiar objects when compared with controls (*P*=0.002, Figure 1d). This deficit was not reversed by FLX administration during adulthood (*P*=0.38; no significant FLX × ND interaction: F_1,44_=2.04, *P*=0.16, Figure 1d).

### ND persistently disrupts mood-like behavior and HPA axis function

**Figure 4:**
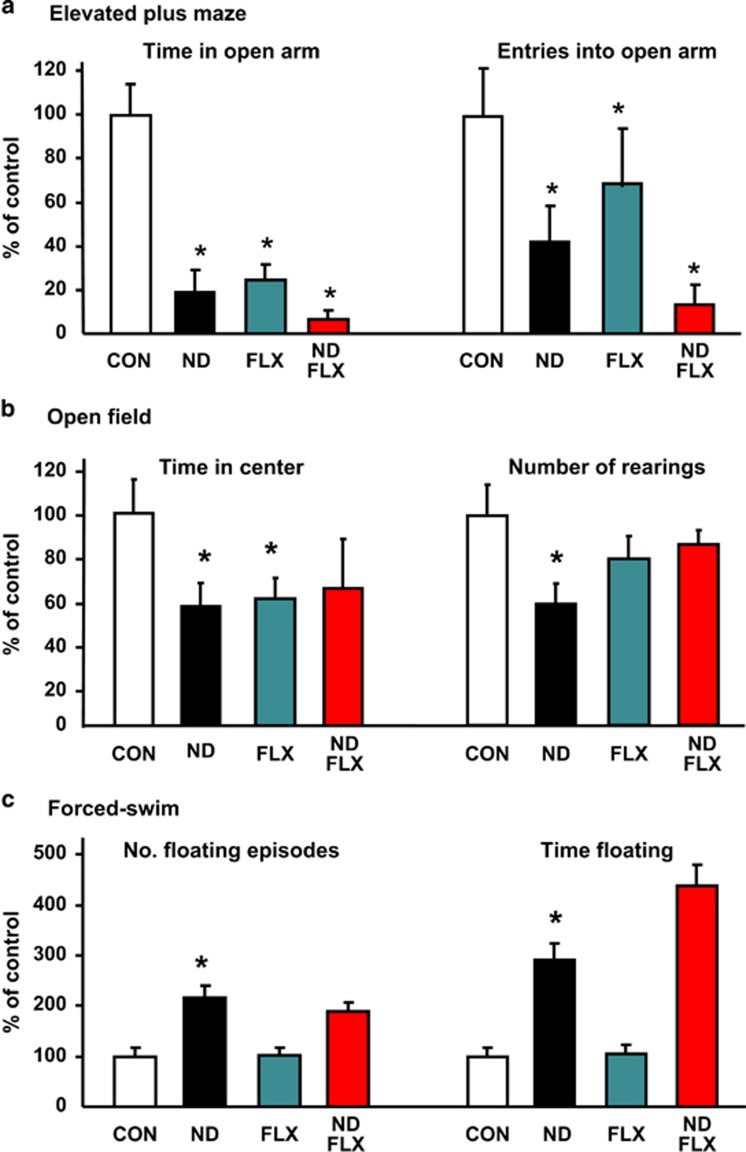
Fluoxetine (FLX) cannot reverse neonatal dexamethasone (ND)-induced increases in stress-coping (depressive-like) behaviors and anxiety. (**a**) Adult rats that had been exposed to ND treatment showed signs of anxiety (less time and less entries into the open arm of an elevated plus maze) as compared with controls (CON) animals, a feature that was not altered by FLX treatment. Note, that FLX itself increased anxiety-like behavior in CON rats. (**b**) ND rats exhibited significantly less rears and spent less time in the central area of an open field arena, indicating their increased state of anxiety. The effects of ND were not counteracted by FLX and rats treated with FLX alone spent less the time in the central area of the arena. (**c**) ND was associated with increased depressive-like behavior, as assessed by the number of floating episodes and time spent floating in the forced-swim test paradigm; FLX did not reverse these effects of ND. All data shown represent mean±s.e.m. (*n*=27, 13, 26, 13 for CON, ND, FLX and ND–FLX groups, respectively). *P<0.05 vs control. *P*<0.05 vs ND.

Emotion, mood and cognition are closely-related behavioral domains,^33, 48, 49^ and comorbidity of mood and anxiety disorders occurs frequently.^3, 32^ ND-treated rats showed increased anxiety-like behaviors in the OF test; specifically, these animals showed fewer rearings (control vs ND: *P*=0.01) and spent less time in the center of the OF arena (F_3,77_=2.21, *P*=0.09; control vs ND: *P*=0.03) (Figure 4a). A similar pattern of behavior was observed when control rats were given FLX (control vs FLX: *P*=0.03); moreover, FLX did not relieve ND-increased anxiety (time in OF arena central area: ND vs ND–FLX: *P*=0.74; rearings: F_3,51_=2.53, *P*=0.07; ND vs ND–FLX: *P*=0.09 (Figure 4a).

ND-treated individuals showed signs of increased anxiety-like behavior in the EPM in terms of time spent in (*P*=0.001), and the number of entries into (*P*=0.05), the open arms of the maze (Figure 4b). FLX elicited anxiety-like behavior in control rats (time in open arms: *P*=0.001; entries into open arms: *P*=0.03) and failed to exert anxiolytic actions in the ND-treated group (the time spent in open arms: F_3,47_=18.15, *P*=0.001; ND vs ND–FLX, *P*=1.00; number of entries into open arms: F_3,47_=3.34, *P*=0.03; ND vs ND–FLX, *P*=0.27); these observations are consistent with previous reports that FLX does not always produce anxiolytic effects in rodents.^50, 51, 52, 53, 54, 55, 56, 57^

Floating, rather than swimming or active struggling, in the forced-swim test serves as an index of reduced ability to ‘switch from active to passive behavior in the face of an acute stressor, aligned to cognitive functions underlying behavioral adaptation and survival’.^39^ Adult rats that had been exposed to ND showed impaired coping behavior; as compared with controls, the ND group displayed a significantly higher number of floating episodes (*P*=0.001) and were immobile (floating) for a significantly longer time (*P*=0.001; Figure 4c). No significant effects of FLX were detected between the number and time of floating episodes in control (time: F_3,44_=26.96, *P*=0.000; control vs FLX: *P*=1.00; number of episodes: F_3,51_=10.55, *P*=0.000; control vs FLX: *P*=1.00) and ND-exposed rats (time: ND vs ND–FLX: *P*=0.16; number of episodes: ND vs ND–FLX: *P*=1.00; Figure 4c).

**Figure 5:**
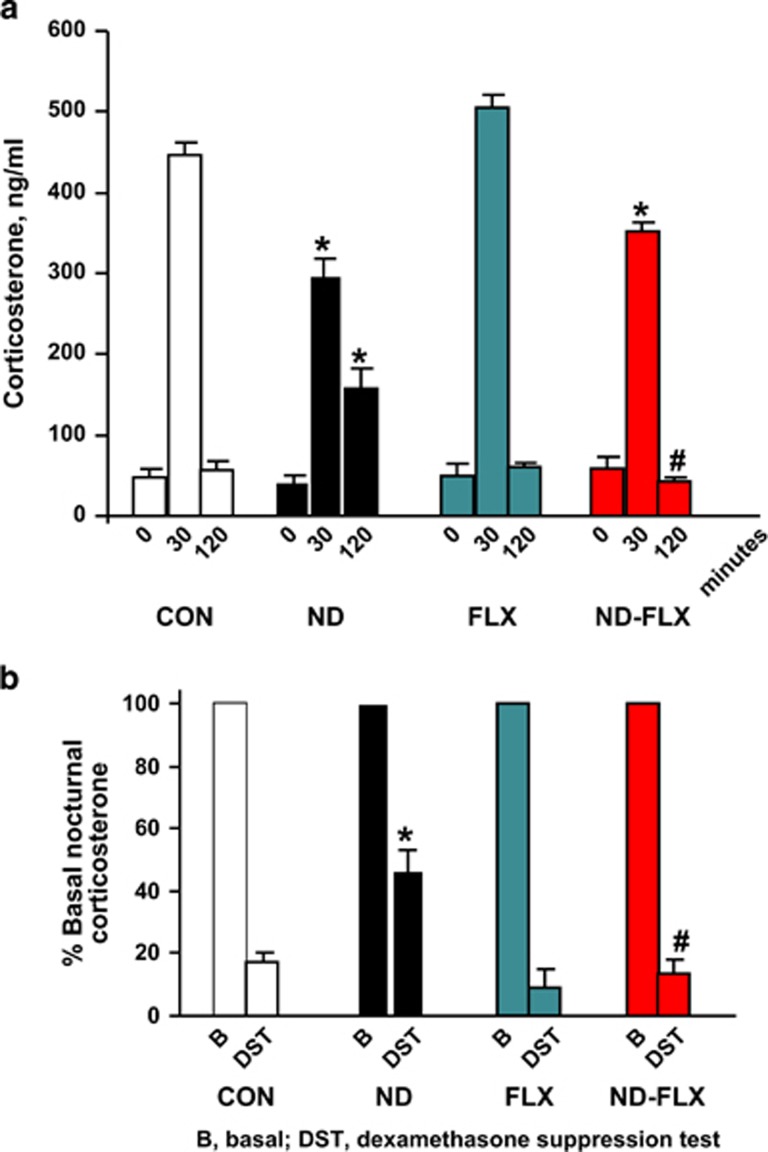
Basal and stress-provoked corticosterone (CORT) secretion. (**a**) Comparison of basal levels of serum CORT and serum CORT concentrations following exposure to an acute stressor after 30 and 120 min. The rate of return to pre-stress levels was used an indicator of glucocorticoid (GC) negative feedback efficacy which is often reduced in depressed patients. (**b**) GC negative feedback efficacy was also evaluated with the dexamethasone suppression test (DST). Animals were held on a 12 l:12D schedule and DEX (10 μg kg^−1^) was injected at ZT6; tail-blood samples were harvested for estimation of CORT at ZT12 when the peak of CORT secretion normally occurs. The latter CORT measurements were compared with levels found in blood samples obtained at ZT12 in each of the treatment groups, and expressed as a percentage. Note that ND animals showed the least suppression of CORT after DEX administration, indicating impaired GC negative feedback. All numerical values are mean±s.e.m. (*n*=27, 13, 26 and 13 for CON, ND, FLX and ND–FLX groups, respectively). *P<0.05 vs the corresponding control value, ^#^*P*<0.05 vs ND-treated counterparts. DEX, dexamethasone; FLX, fluoxetine; ND, neonatal DEX.

Impaired GC negative feedback mechanisms and hypersecretion of GC is a common feature in depression in humans^29, 58^ and in animal models of the disease.^59^ In this study, serum corticosterone levels did not differ between control and ND-treated animals during the daily nadir in the activity of the HPA axis (*P*=0.66; Figure 5a); this finding indicatings that ND did not suppress adrenocortical secretion under basal conditions. On the other hand, ND-exposed rats displayed discrepant GC secretory responses when confronted with an acute stressor (Figure 5a); despite their significantly higher resting levels of corticosterone (*P*=0.001), ND-treated rats responded to acute stress with a sluggish and significantly blunted corticosterone response (*P*=0.001). Further, results of a DST showed that ND-treated animals are impaired in terms of GC negative feedback: whereas the daily nocturnal rise in corticosterone secretion was fully suppressed by DEX in control animals, DEX only partially suppressed corticosterone secretion in ND-treated animals (*P*=0.001; Figure 5b).

Although FLX did not affect baseline corticosterone secretion (F_3,57_=0.33, *P*=0.8; Figure 5a), the antidepressant improved GC negative feedback after exposure of ND-treated rats to an acute stressor (at 120 min-post stress: F_3,61_=13.2, *P*=0.000; control vs ND–FLX: *P*=0.48; ND vs ND–FLX: *P*=0.001; Figure 5a). Moreover, FLX treatment during adulthood resulted in improved GC feedback efficacy in ND-exposed animals (F_3,53_=10.06, *P*=0.001; control vs ND–FLX: *P*=1.0; ND vs ND–FLX: *P*=0.001; Figure 5b).

## Discussion

Neuronal turnover in the hippocampus is a dynamic process which includes neurogenesis and apoptosis in the SGZ of the dentate gyrus.^4^ The hippocampus undergoes its most dynamic structural organization during early postnatal life^60^ but new granule neurons continue to be generated throughout life, albeit at progressively lower rates as individuals age.^61^ Abundant evidence indicates the importance of neurogenesis and apoptosis in modulating preexisting neurocircuits.^62, 63^ Apoptosis has an important role in the control of the size of the granule neuron population. Although post-mitotic neurons are endowed with robust anti-apoptotic mechanisms, these can be subjugated by GC^3, 64, 65^ and aging,^63^ resulting in significant attrition of the granule cell layer. In light of demonstrations that the (finite) population of NPC in the dentate gyrus are vulnerable to GC-induced apoptosis,^30^ the present study was designed to examine the functional consequences (and reversibility) of ND exposure. This was considered important because DEX appears in the *WHO Model List of Essential Medicines* for children (http://www.who.int/medicines/publicati-ons/essentialmedicines/EMLc_2015_FINAL_amended_AUG2015.pdf?ua=1).

The results obtained in the present study show that ND has a persistent negative impact on the neurogenic capacity of the hippocampus. In ND-treated animals, the reduction in the hippocampal pool of NPC (Sox2+ cells) and the number of actively dividing NPC (Ki67+/Sox2+ cells) in ND-treated animals is accompanied by shrinkage of the SGZ; however, it is important to note that some cells may escape the actions of DEX because they do not express GR.^30, 66^ As the inhibitory effect of DEX on neuronal proliferation can be spontaneously reversed, the persistent impairment of the neurogenic pool is most likely due to the loss of neural progenitors during neonatal life. The finding that ND-exposed adult rats display reduced GCL volumes and deficits in spatial and object-recognition memory confirms earlier suggestions that neurogenesis is important for the manifestation of hippocampus-dependent behaviors.^5, 46, 62, 67^

GC stimulate apoptosis of hippocampal neurons^41, 42^ and of NPC within the SGZ.^30, 44^ In the present study, adult ND-treated rats did not display increased hippocampal levels of cleaved caspase 3, a marker of active apoptosis. However, ND treatment was associated with higher expression ratios of pro-apoptotic (Bax) vs anti-apoptotic (Bcl-2 and Bcl-xl) proteins, indicating greater vulnerability of hippocampal cells to apoptosis.^30, 45^ As ND treatment depletes the NPC pool through apoptosis,^30^ and astrocytes are resistant to DEX-induced apoptosis,^40^ we suggest that the cells displaying signs of vulnerability to apoptosis during adulthood are likely to be mature granule neurons.

The hippocampus occupies a key position in the central regulatory cascades that serve to restrain GC secretion following stress.^68^ Failure to curtail GC secretion in a timely manner compromises stress-coping ability and may trigger the onset of depression and anxiety,^29, 58, 69^ cognitive dysfunction^3, 48, 49^ and possibly, Alzheimer disease pathology.^37, 70^ The association between ND treatment and impaired GC negative feedback, together with the behavioral phenotype observed, reinforces the view that exposure to stress or GC during early life can lead to protracted/lifelong disruption of mental functions.^70, 71^ Interestingly, we found that administration of the antidepressant FLX during adulthood reverses the ND-triggered inhibition of mitosis in the dentate gyrus and the disruptive effect of ND on GC negative feedback; however, FLX failed to rescue ND-induced impairments in memory and adaptive behavior. This observation, which is consistent with a previous report that adult neurogenesis is required for FLX to normalize HPA axis activity,^72^ also suggests that a mechanism, other than proliferation, contributes to the overall therapeutic effects of FLX, a possibility worthy of further investigation.

Consistent with the results of studies with various antidepressants,^33, 73, 74^ FLX was found to stimulate mitosis within the dentate gyrus of ND-exposed animals, but not control animals. Strikingly, however, FLX did not restore the NPC pool in ND-treated animals, indicating that chronic FLX treatment in adulthood is not sufficient to rescue the effects of ND treatment. GC receptors, which mediate the apoptotic effects of DEX, are expressed by both quiescent neural progenitors and amplifying neural progenitors in the SGZ.^30^ Interestingly, FLX application during adulthood only promotes division of amplifying neural progenitors;^75^ this offers a plausible explanation for why FLX treatment stimulates neurogenesis in the absence of a concomitant increase in the NPC pool. In this context, it is important to recall that NPC have a limited capacity for self-renewal and that neurogenesis wanes with increasing age;^76, 77^ accordingly, accelerated age-related deterioration of hippocampal functions that depend on neurogenesis would be a predictable outcome of ND treatment.

Given that the hippocampus is reciprocally connected with the amygdala, bed nucleus of the stria terminalis, nucleus accumbens and medial prefrontal cortex, it is not surprising that neurogenesis in the hippocampus has an impact on mood and emotion.^6, 7, 8, 9^ The results of the present study show that both, ND and FLX induce hyperanxiety; they also show that FLX does not act as an anxiolytic when administered to ND-exposed animals. Interpretation of these findings is challenging because of the highly variable direction of effects of FLX on anxiety levels in animals: FLX has been variably described to have no effects,^51, 52^ anxiolytic effects^33, 53^ or anxiogenic effects^54, 55, 56, 57^ in animals; these disparate reports most likely reflect differences in animal strain,^54^ age^78^ and/or experimental conditions such as handling.^79^

In summary, our results demonstrate that ND impairs a variety of hippocampus-dependent functions, ranging from neuroendocrine homeostatic mechanisms to the regulation of affective and cognitive behaviors. These impairments occur contemporaneously with the depletion of the hippocampal NPC pool, persistent inhibition of hippocampal neurogenesis and, increased vulnerability of hippocampal neurons to apoptosis. As FLX administration enhances the survival of hippocampal neurons, we suggest that timely application of antidepressants may help rescue at least some of the behavioral functions that are lost following ND-induced reductions in the neurogenic capacity of the hippocampus. Our findings that early postnatal life represents a phase during which NPC are particularly sensitive to DEX lend support to the American Academy of Pediatrics’ policy on the use of glucocorticoids in pediatric practice.^18^ On the other hand, and notwithstanding the limitations of extrapolating results from one species to another, it deserves mentioning, (i) that neurogenesis is only transiently inhibited when DEX is administered to adult rats,^64^ and (ii) that prenatal DEX does not persistently impair the volume and proliferative and differentiation capacity of the non-human primate hippocampus.^80^ Therefore, exploration of the existence of developmental windows during which DEX therapy may be safely applied will be a worthwhile pursuit in future investigations.
