# Supplementary material for: Antidepressant responsiveness in adulthood is permanently impaired after neonatal destruction of the neurogenic pool
Source: Transl Psychiatry. 2017 Jan 3;7(1):e990–. doi: 10.1038/tp.2016.255 (PMC5545723; doi:10.1038/tp.2016.255)
Supplement: Supplementary Materials [file tp2016255x1.docx]

# SUPPLEMENTAL MATERIALS

**
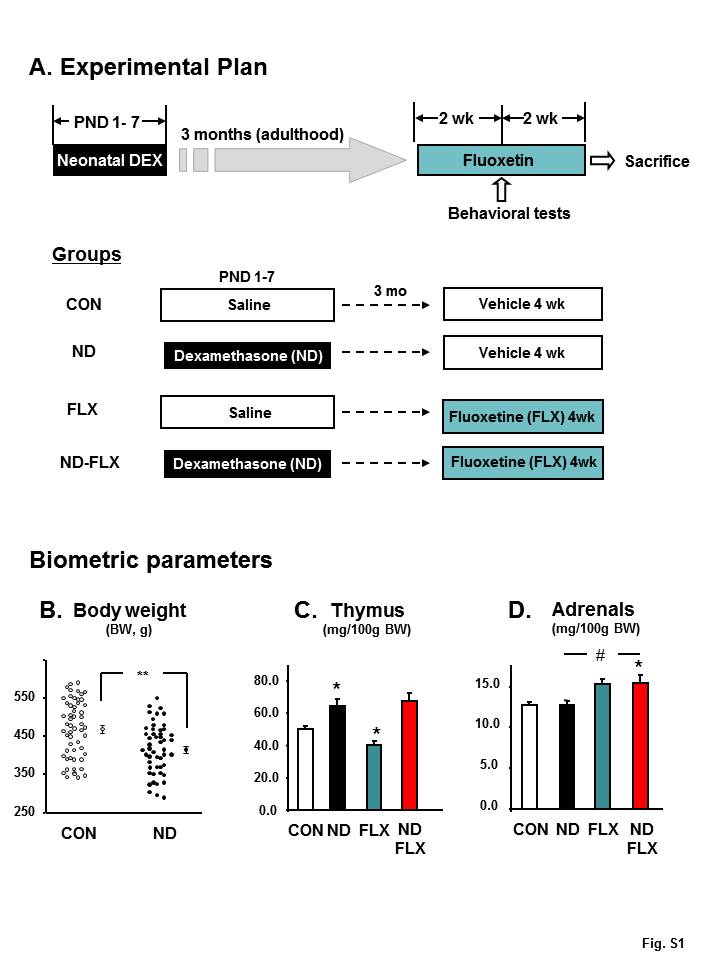
**

**Figure S1. A. Experimental design.** From postnatal (PND) 1 to PND 7, rat pups received a daily injection of saline or a tapering dose (PND 1-3: 200 µg/kg; PND 4-7: 100 µg/kg) of dexamethasone (neonatal dexamethasone, ND). At the age of 3 months, subgroups of control (CON) and ND-treated rats were treated with vehicle or fluoxetine (FLX, 10 mg/kg, daily) for 4 weeks. Behavioral testing was performed during the last 2 weeks of the latter treatment phase. As shown, the experiment consisted of 4 treatment groups. A number of biometric parameters were monitored as an index of treatment efficacy just before sacrifice. **B.** Body weight was significantly reduced in ND-treated animals (n = 55), as compared to CON animals (n = 50). **C.** Relative thymus weights in CON (n = 12), ND (n = 13), FLX (n = 12) and ND-FLX (n=12) are shown. ND and FLX rats, respectively, displayed significantly higher and lower thymus weights as compared to CON rats. **D.** Relative adrenal weights in CON (n = 12), ND (n = 13), FLX (n = 12) and ND-FLX (n=12) are depicted. Adrenal weights were significantly higher in the ND-FLX group than in CON and ND-treated animals. All numerical values are mean + standard error of the mean (SEM). * indicates p <0.05 *vs.* CON; # denotes p < 0.05 *vs.* ND-treated group.

**
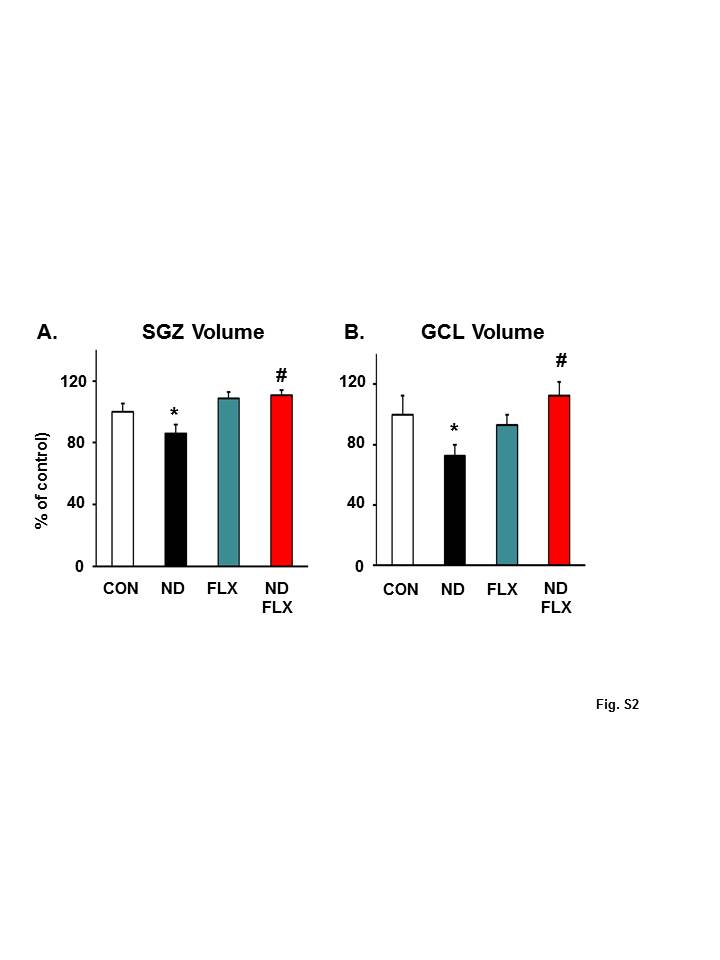
**

# Figure S2. Hippocampal granule cell layer and subgranular (neurogenic) zone volumes after ND and FLX treatment. Rats were treated according to the schedule depicted in Fig. 1, and subgranular (neurogenic) zone (SGZ, A) and hippocampal granule cell layer (GCL, B) volumes were estimated using stereology after sacrifice when animals were aged 4 months. Data are shown for CON, ND, FLX and ND-FLX- (n = 8 per group) treated animals. All numerical values are shown as means + SEM. * denotes p < 0.05 *vs.* CON; # indicates p < 0.05 *vs.* ND.

**
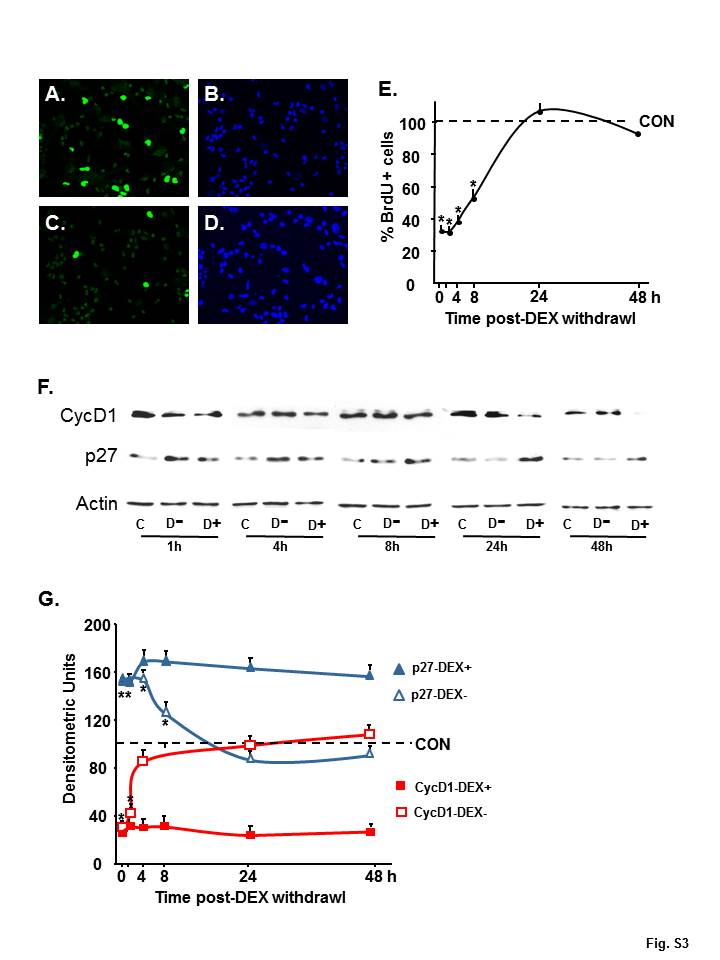
**

# Figure S3. Restoration of proliferative capacity following washout of dexamethasone (DEX) from primary hippocampal cultures. Primary hippocampal cultures, derived from postnatal rats aged 4 days, in which ~75% of cells are neural precursor cells (NPC) or immature neurons (Yu et al., 2010), were treated with DEX (10-6 M) for 7 days *in vitro* (DIV). After 24 h, DEX was washed out and the recovery of proliferation capacity was evaluated at different intervals by immunocytochemistry for 5-bromo-2’-deoxyuridine (BrdU). A-D, depict representative images of BrdU- stained cells (A, C) and Hochest 33342 (nuclear counterstain, B, D) in control (A, B) and 24 h DEX-treated (C, D) cells. BrdU incorporation was assayed after cells had been exposed to BrdU (20 µM) for 4 h before termination of the experiment. A quantitative analysis of the percentage of BrdU-immunopositive cells at different time points after DEX withdrawal is presented in panel E. The dynamic regulation of key regulators of the G1 phase of the mitototic cycle at different times after DEX withdrawal is shown in representative Western blots (F) and semi-quantitative data from such immunoblotting studies (n = 4) are shown in panel G. Scale bar = 50 µm. Numerical data represent mean + SEM. * p < 0.05 indicates significant differences between results from DEX withdrawn samples *vs.* controls at the corresponding time point.
